# Supplementary material for: Rephine.r: a pipeline for correcting gene calls and clusters to improve phage pangenomes and phylogenies
Source: PeerJ. 2021 Aug 6;9:e11950. doi: 10.7717/peerj.11950 (PMC8351571; doi:10.7717/peerj.11950)
Supplement: Supplemental Information 1 — Red dots correspond to cases with both low overlap and low sequence identity, indicating the likeliest fragmented gene calls. [file peerj-09-11950-s001.pdf]

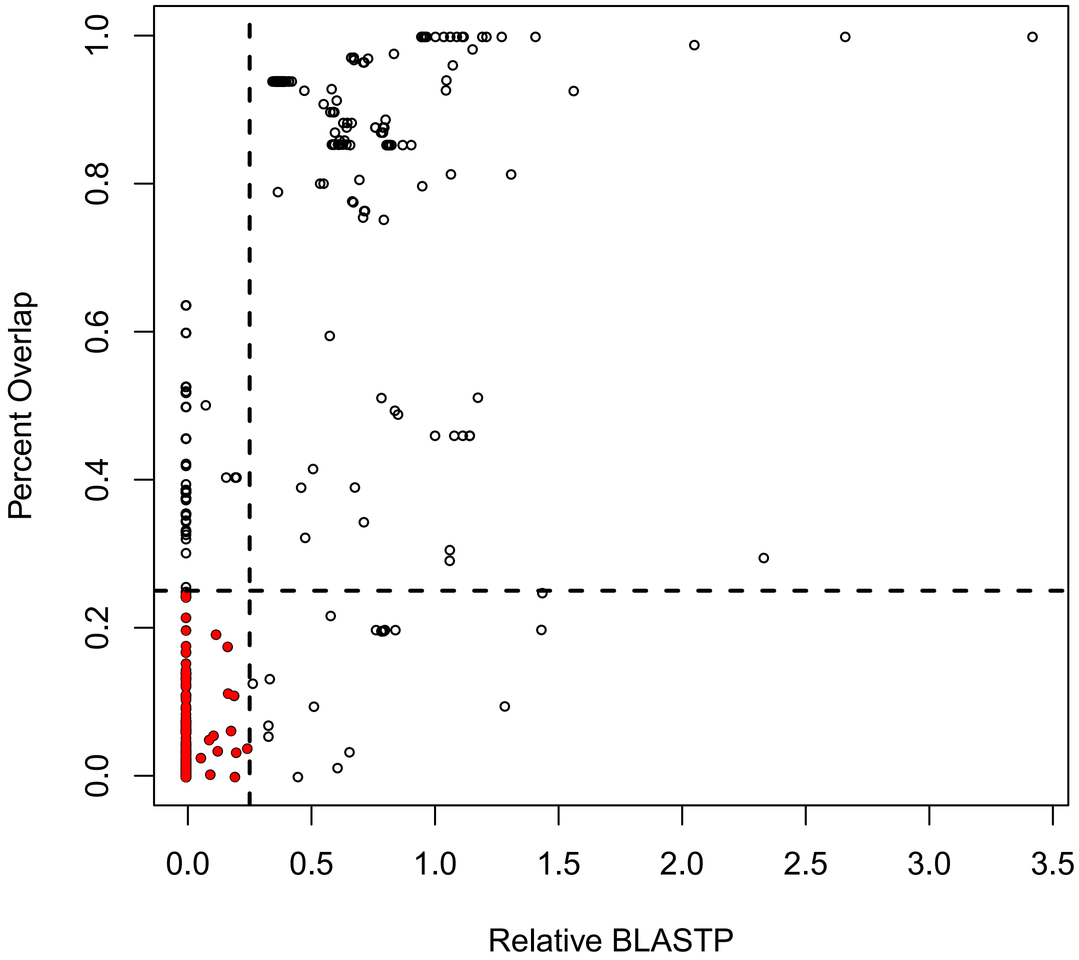

Supplemental Figure 1. Relationship between pairwise overlap of aligned positions and relative bit scores of potential paralogs. Red dots correspond to cases with both low overlap and low sequence identity, indicating the likeliest fragmented gene calls.
