# Supplementary material for: Rephine.r: a pipeline for correcting gene calls and clusters to improve phage pangenomes and phylogenies
Source: PeerJ. 2021 Aug 6;9:e11950. doi: 10.7717/peerj.11950 (PMC8351571; doi:10.7717/peerj.11950)
Supplement: Supplemental Information 2 — The type phages T3 and T7 are shown in bold. Bootstrap support is shown by coloring branches preceding nodes, with low support (from 0 to 70) ranging from white to red. [file peerj-09-11950-s002.pdf]

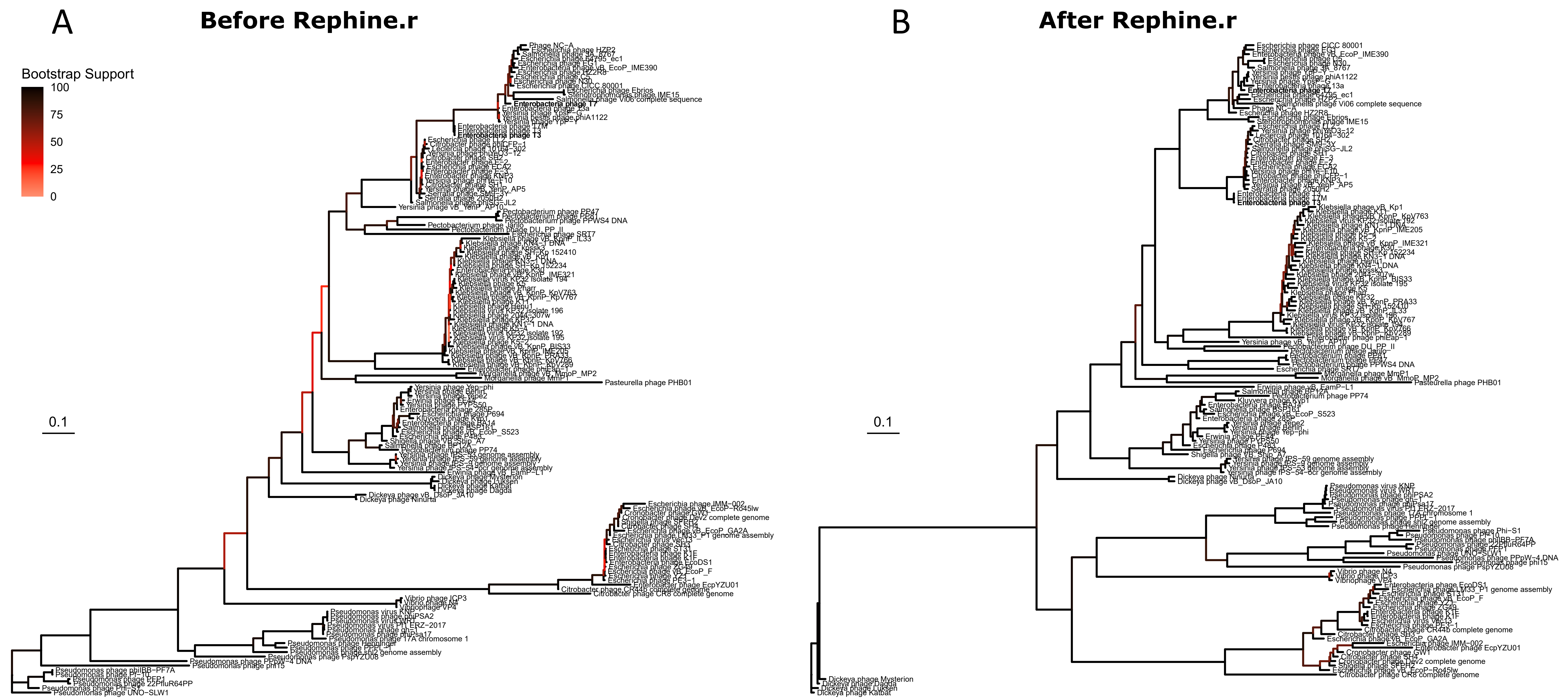

Supplemental Figure 2. Phylogeny of Studiervirinae before and after running Rephine.r, with all tips labeled by the corresponding RefSeq phage name. The type phages T3 and T7 are shown in bold. Bootstrap support is shown by coloring branches preceding nodes, with low support (from 0 to 70) ranging from white to red.
