# Supplementary material for: Rephine.r: a pipeline for correcting gene calls and clusters to improve phage pangenomes and phylogenies
Source: PeerJ. 2021 Aug 6;9:e11950. doi: 10.7717/peerj.11950 (PMC8351571; doi:10.7717/peerj.11950)
Supplement: Supplemental Information 3 — The type phage PB1 is shown in bold. Bootstrap support is shown by coloring branches preceding nodes, with low support (from 0 to 70) ranging from white to red. Note: an outlier genome ( NC_009015) was dropped from the tree to enable visualiza [file peerj-09-11950-s003.pdf]

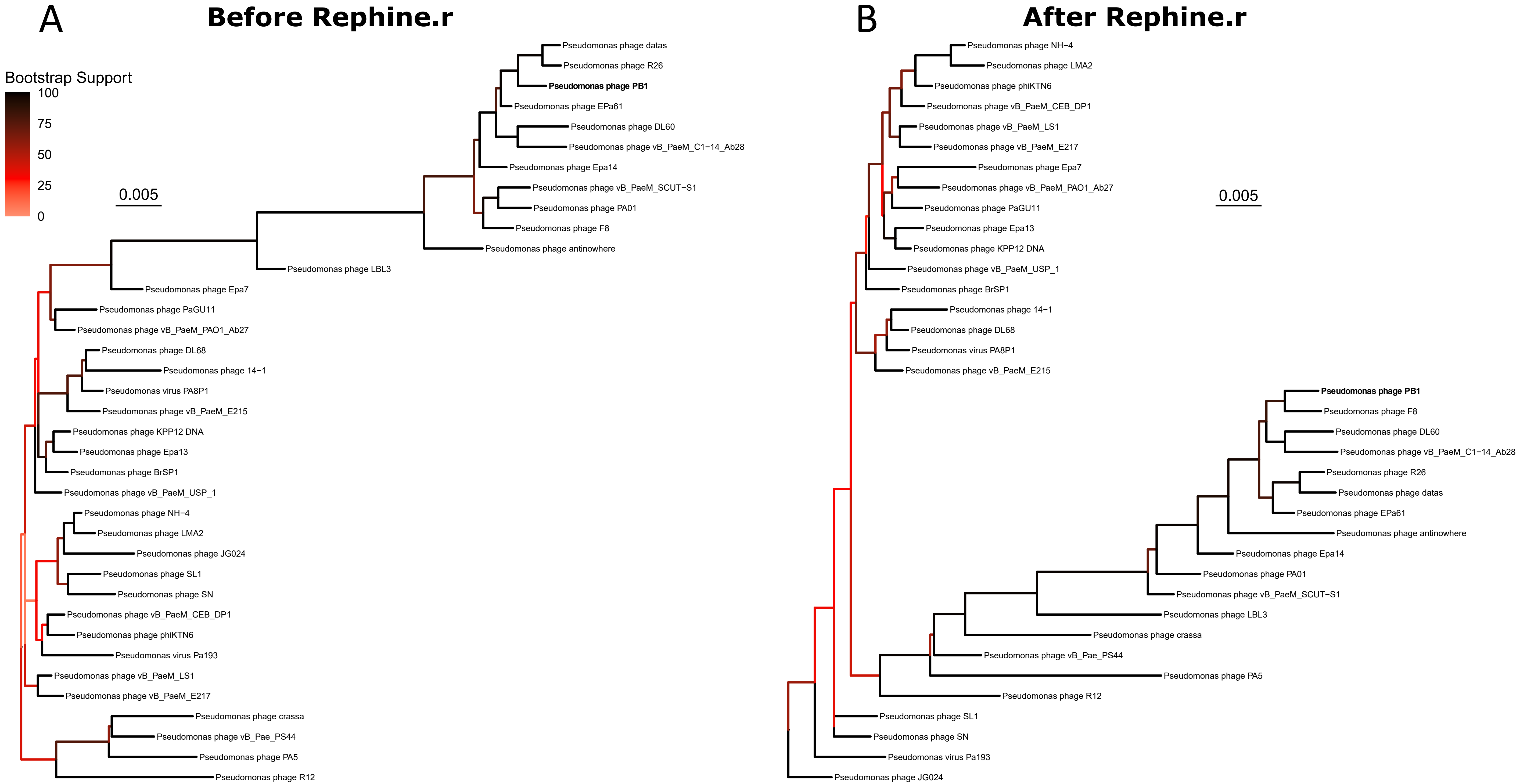

Supplemental Figure 3. Phylogeny of Pbunaviruses before and after running Rephine.r, with all tips labeled by the corresponding RefSeq phage name. The type phage PB1 is shown in bold. Bootstrap support is shown by coloring branches preceding nodes, with low support (from 0 to 70) ranging from white to red. Note: an outlier genome (NC_009015) was dropped from the tree to enable visualization of the extremely short branches.
