# Supplementary figures and images for: Rephine.r: a pipeline for correcting gene calls and clusters to improve phage pangenomes and phylogenies
Source: PeerJ. 2021 Aug 6;9:e11950. doi: 10.7717/peerj.11950 (PMC8351571; doi:10.7717/peerj.11950)

## Before Rephine.r

## After Rephine.r

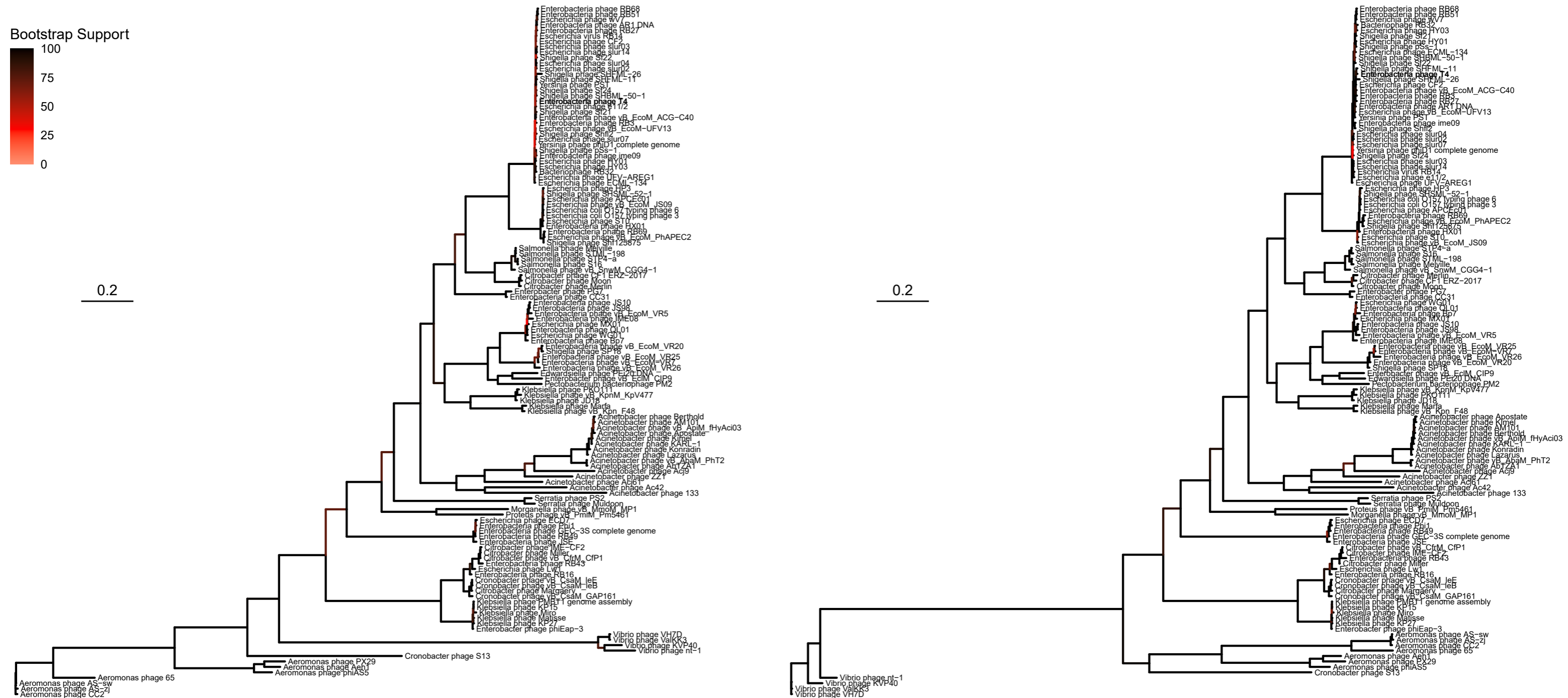

Supplement: Supplemental Information 4 — The type phage T4 is shown in bold. Bootstrap support is shown by coloring branches preceding nodes, with low support (from 0 to 70) ranging from white to red. [file peerj-09-11950-s004.pdf]
